# Alleviating Exhaustion: The Mediating Role of Well-Being in the Relationship Between Research Disengagement and Burnout Among Tunisian University Lecturers

**DOI:** 10.62641/aep.2419

**Published:** 2026-10-15

**Authors:** Abu Elnasr E. Sobaih, Hassane Gharbi

**Affiliations:** ^1^Management Department, College of Business Administration, King Faisal University, 31982 Hofuf, Al-Ahsa, Saudi Arabia; ^2^Management Department, Faculty of Economics and Management of Nabeul, Carthage University, 8000 Nabeul, Tunisia

**Keywords:** disengagement at work, burnout, well-being at work, university lecturers, higher education

## Abstract

**Background:**

This study examines the direct influence of research-focused work disengagement on general academic burnout and explores the underlying psychological mechanisms by testing the indirect link through teaching-specific well-being.

**Methods:**

A questionnaire was distributed to 17 institutions (universities, faculties, institutes, and schools), following the ethical standards, located in Tunis, Jendouba, Nabeul, Sousse, Mahdia, and Sfax. A total of 950 university lecturers of Tunisian nationality completed a questionnaire. The study was conducted over a period of five months from the beginning of 2025, and at the end of this period, we collected 636 usable questionnaires, representing a response rate of approximately 66.95%. Because participation relied on convenience sampling and institutional access permission, the sample presents demographics skews including 75% female representation and an 80% non-publishing rate, introducing potential self-selection bias that limits broad population generalization. Data analysis was performed using Structural Equation Modelling (SEM) via AMOS 25 to evaluate the hypothesized paths and estimate indirect mediation effects via a robust bootstrapping procedure.

**Results:**

The findings supported the initial hypotheses. Specifically, research disengagement significantly and negatively affects general academic burnout, while significantly and positively predicting teaching-specific well-being. Additionally, teaching-specific well-being was found to significantly and negatively influence burnout. When introduced into the path model, teaching-specific well-being exhibited a significant indirect association, indicating that it exerts a partial mediating effect on this relationship among Tunisian teacher-researchers.

**Conclusions:**

Teaching-specific well-being is associated with a partial mediating role in linking research disengagement to general academic burnout among university lecturers. These findings offer important practical and theoretical implications for academic institutions and researchers seeking to mitigate burnout and support faculty well-being.

## Introduction

The academic world is built on an unspoken foundation; excellence stems from the sum of teaching and research. To create value and consolidate academic identity, modern universities must ensure a close relationship between scientific output and teaching [1, 2]. It should be noted that teaching is considered effective only if it yields two results. First, it promotes students’ academic success by optimizing their knowledge, skills, and educational qualifications [3]. Second, it confirms their prospects through unequivocal employability, while research productivity is measured by the number of publications [3, 4, 5]. Rowland [6] explored the link between teaching and research activities through qualitative research based on informal meetings with 12 heads of department at British universities. The results showed that both academic activities were in demand. Nevertheless, research has enjoyed a prestigious position within institutions. Furthermore, a qualitative study conducted in New Zealand has revealed the need to prove a dialectical link between teaching and research. Indeed, engagement in research further enhances teaching, and serious involvement in which in turn stimulates the need to conduct research [7]. Furthermore, in the United States, quantitative research was conducted on a national sample, collecting responses from 3968 professors with the aim of studying academic work within the American university system in specific departments. The study found that research and teaching are not a single dimension of university investment but are diverse with different dimensions [8].

Within the Tunisian public university framework, the institutional landscape imposes unique statutory demands on academics, which frequently leads to structural strains between teaching mandates and research expectations. Specifically, the Tunisian Decree No. 93-1825, year 1993, specifies that teaching and research staff are required to add to the growth and transmission of knowledge through initial and continuing training by providing supervision and participating in the improvement of teaching methods. In addition, they are required, among other things, to contribute to scientific research activities by contributing to their development. In the absence of scientific research activities, legislation blocks the career of the teacher-researcher while providing disciplinary measures in the event of proven failure to comply with statutory obligations. However, a distinct misalignment exists between statutory text and actual operational enforcement in the local context. In practice, only professional deadlock, manifested by proven career stagnation, is operational in the Tunisian context. Nevertheless, and until proven otherwise, the disciplinary measures referred to in the above text of the law seem to play the role of a utopian ghost, referring precisely to the concept of a law that has remained a dead letter, having to date not taken any effective disciplinary action against a teacher-researcher showing indolence towards scientific research.

Thus, based on the above, we can state our problem: the conventional assumption that research disengagement inevitably leads to burnout among university lecturers is confronted with a rather enigmatic and even suspicious reality on the ground, where the existence of a law that remains a dead letter, protecting lecturers from effective sanctions, suggests a counterintuitive dynamic.

Preliminary pilot observations and anecdotal evidence from Tunisian faculty boards suggest that a substantial proportion of lecturers deliberately pivot away from unrewarded research activities to conserve emotional resources, yet empirical validation of these constrained institutional adaptations still lacking in the literature. To address this gap, this study systematically pursues two explicit research objectives: (1) to determine the direct and indirect impacts of research disengagement on teacher-researcher burnout within public institutions, and (2) to rigorously evaluate whether work-related well-being performs a partial or total mediation role in this process. Accordingly, the central research question guiding this inquiry is: To what specific degree does a lecturer’s disengagement from mandatory research duties associated with their professional well-being and subsequent experience of academic burnout?

Drawing upon Conservation of Resources (COR) theory, we conceptualize research disengagement not merely as a passive opposite of engagement, but as a domain-specific form of psychological withdrawal and selective resource allocation. We define disengagement at work as a psychological or behavioural state manifested by a decline in an individual’s emotional, cognitive, and physical investment in their work activities or organization [9]. As such, disengagement at work constitutes a breach of contractual and/or organizational obligations. Maslach *et al*. [10] explain this organizational phenomenon as reduced participation and inefficiency in terms of employment and organization. Furthermore, disengaged and unenthusiastic employees who do not participate in collective efforts are likely to negatively affect organizational performance [10]. In proven cases, disengagement can lead to high staff turnover [11]. In the context of our research, disengagement among teacher researchers occurred when they focused all their energy on teaching and ignored scientific research. Tenured and protected by a law that remains a dead letter, university lecturers find themselves unable to fulfil an obligation that is supposed to be of paramount importance, namely scientific output, which is no longer a priority for them.

Burnout refers to the failure of meaning at work [12]. It is characterized by a lack of enthusiasm and a disconnect from organizational goals [13] and causes employees to perform below average [14, 15, 16]. Burnout occurs when psychological balance is disrupted, and as a result, well-being at work is compromised. This state of suffering stems from repetitive stress, meaning that they cannot deal with it, which would lead to demotivation and frustration [17].

Studies have also examined the relationship between disengagement at work and burnout. Examples include Harris [18], Basińska and Gruszczyńska [19], and Bakker and de Vries [20], which ultimately confirmed this association. This relationship was further reinforced by Cole *et al*. [21], who, based on thirty-seven publications, confirmed a substantial correlation between disengagement and professional burnout. This finding was supported by the alarming statistical data presented by Bakker *et al*. [22], who revealed that one out of five workers was highly engaged in their jobs, resulting in high levels of professional exhaustion.

While standard Job Demands-Resources (JD-R) models assert that detachment reflects a state of severe exhaustion [20], contemporary perspectives in higher education across developing nations suggest that selective disengagement can act as a strategic, self-protective mechanism against institutional overload. In African and emerging-economy academic environments, faculty members frequently navigate severe institutional constraints, including structural underfunding, high student-to-teacher ratios, and an acute lack of research infrastructure [23]. Although general theory attests to a positive relationship between disengagement and burnout, we argue that in the Tunisian public university context, job security alters this path. Based on the premise that insecurity motivates effort in hospitality context [24], strong statutory protection can lead to a protective reduction of research activity. Because there are no real consequences for neglecting research, the pressure disappears, effectively insulating the individual from performance-related stress [20]. Thus, we suggest:

H1- Research-focused work disengagement is negatively associated with general academic burnout.

Undoubtedly, disengagement at work is detrimental to companies concerned about their financial health. Statistics prove this, revealing that a large proportion of the workforce is disengaged [25], which causes excessive financial costs for organizations [26]. From this perspective, well-being at work has not been more vital than it is today [27]. Disengagement occurs when employees do not show enthusiasm for or commitment to their jobs [10, 28]. In this regard, Wollard [29] adds that “disengagement manifests itself through individuals disconnecting from their professional roles in order to safeguard themselves physically and emotionally from objective or subjective threats” (p. 528).

According to Fredrickson’s Broaden-and-Build theory [30], the experience of positive emotional states expands an individual’s personal resources, whereas persistent negative states diminish well-being. Concurrently, researchers have noted that when specific work domains (such as mandatory publication demands) become primary sources of unmitigated anxiety, intentionally withdrawing from that specific domain allows individuals to redirect their emotional resources to domains with immediate, positive reinforcement, such as interactive classroom teaching, thereby restoring immediate work-related well-being. Although standard applications of Broaden-and-Build theory assume that overall organizational detachment erodes psychological capital, selective withdrawal from a highly stressful, unrewarded research environment can act as an active resource-preservation strategy under COR theory. This alternative path allows faculty members to avoid chronic frustration and maintain an overall positive orientation toward their daily teaching roles. While general corporate studies indicate that work engagement is a positive predictor of employee well-being [31, 32, 33], the compartmentalized nature of academic tasks allows for a distinct configuration. In this specific context, our hypothesis is as follows:

H2- Research-focused work disengagement is positively associated with workplace self-actualization well-being.

As paradoxical as it may seem, well-being is the opposite of burnout [9, 34, 35], and the two concepts are usually measured using the same measurement tool by researchers. Defining teacher well-being is an additional complex and multidimensional task [36]. Well-being drives employee effectiveness [37]. It highlights a holistic perspective, encompassing both the physical condition and professional and personal satisfaction of employees [38]. Furthermore, it stimulates individual, organizational, and societal productivity [39, 40, 41]. Given the significant increase in burnout in the academic world, promoting the well-being of academic staff has become a key challenge that no university can disregard [42]. Furthermore, teacher-researcher burnout negatively influences student engagement [43]. This observation reinforces the idea of reconsidering teacher researchers’ well-being as an essential prerequisite for ensuring educational success and student well-being [44].

Schaufeli and Enzmann [45] described burnout as a complex state of mind characterized by persistent and negative work-related feelings in individuals. It is not limited to fatigue but also includes feelings of emotional distress [46, 47], low self-efficacy [48, 49, 50], disinterest in work [51] and the development of dysfunctional behaviors in the workplace [45]. A survey conducted on police officers showed a significant negative relationship between well-being at work and burnout. Burnout had the highest contribution, followed by job satisfaction, work engagement, and occupational stress, indicating that the development of well-being at work contributes to a significant reduction in burnout among police officers [52]. Thus, we suggest:

H3- Workplace self-actualization well-being is negatively associated with general academic burnout.

H4- Workplace self-actualization well-being mediates the association between research-focused work disengagement and general academic burnout.

Fig. 1 summarizes the relationship and offers the research conceptual framework.

**Fig. 1.**
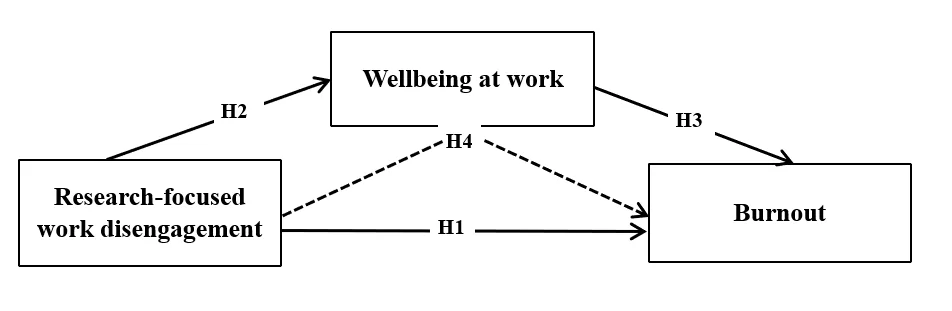
**The proposed model**.

## Methods

### Population, Sampling and Measurement

To test the hypothesized paths, an empirical study was implemented using a cross-sectional convenience sampling design across public higher education structures. Because participation was voluntary and relied on institutional access permissions, the sample presents a demographic distribution where female lecturers represent 75% of respondents (Table 1). This gender imbalance warrants explicit note, as it reflects a higher response propensity among female faculty members within the participating departments, which deviates from the wider historical over-representation of males in senior ranks within Tunisian public universities. Furthermore, 80% of respondents reported no research output in the past five years, indicating that the sample overrepresents faculty with negative attitudes or disengagement toward research, which introduces self-selection bias.

**Table 1.** **Profile of participants (N = 636)**.

Items	Class	Quantity	Proportion %
Gender	Male	159	25
	Female	477	75
Marital status	Married	490	77.04
	Single	146	22.96
Years of seniority	Less than 10 years	46	7.23
	Between 11 and 20 years	200	31.45
	More than 20 years	390	61.32
Age	< 30	77	12.11
	30–40	151	23.74
	41–50	320	50.31
	>50	88	13.84
Professional grade	Assistant	55	8.65
	Senior Assistant	422	66.35
	Senior Lecturer	102	16.04
	Full Professor	57	8.96
Do you ever do scientific research?	Yes	127	19.97
	No	509	80.03
If so, how many articles have you published in the last five years?	Between 1 and 5 articles	73	57.48
	Between 6 and 10 articles	43	33.86
	Between 11 and 20 articles	11	8.66
	More than 20 articles	0	0
Total		636	100

The questionnaire was distributed to a convenience sample of 17 institutions (universities, faculties, institutes, and schools) located in Tunis, Jendouba, Nabeul, Sousse, Mahdia, and Sfax. A total of 950 university lecturers were contacted who are in higher education and under the control of Decree No. 93-1825, Year 1993, yielding 636 usable questionnaires over a five-month fieldwork period in 2025, which represents a response rate of 66.95% valid for analysis. Other forms that are collected but were incomplete and not valid for analysis were archived securely to protect the privacy of respondents.

To measure the core constructs, established scales from the literature were operationalized and adapted. Work disengagement was examined using nine items adapted from Schaufeli and Bakker’s [53] Utrecht Work Engagement Scale (UWES). While the original UWES utilizes a 0–6 seven-point frequency scale, and the Copenhagen Burnout Inventory (CBI) utilizes a 0–100 percentage-based five-level scoring system, all scales in this study were adapted into a uniform 1–5 Likert-type scale (1 = strongly disagree, 5 = strongly agree) to maintain survey consistency for respondents—a methodological adaptation that is acknowledged as a limitation. Because the UWES natively tracks active engagement, these items were deliberately adapted by reversing their directional wording to directly track research-specific disengagement (e.g., “When it comes to research, I don’t feel full of energy”). While psychometric research often highlights an ongoing debate regarding whether engagement and disengagement are opposites or separate constructs [54], the item-reversal strategy was utilized here to specifically isolate faculty members’ psychological detachment from the research component of their roles. Burnout was measured via the seven-item scale developed by Kristensen *et al*. [55], and well-being at work was assessed through the four-item scale by Bartels *et al*. [56], evaluated specifically as intrapersonal workplace self-actualization well-being, avoiding the broad general well-being label. It is theoretically crucial to note that the measurement items are domain-stratified: the disengagement items (D1–D8) explicitly reference research withdrawal, whereas the well-being items (W17–W20) explicitly capture emotional stimulation derived from teaching activities (see **Supplementary material**). The burnout scale contains items that span across both domains, allowing the model to capture how domain-specific research detachment and teaching satisfaction interactively shape general professional exhaustion. The scale was disrupted in both English and French language as participants are aware of the two languages. Back-to-back translation and was double checked by two bilingual experts.

The average of answers ranged from 1.73 to 4.55 with standard deviation (SD) ranged from 0.773 to 0.926 (see Table 2), confirming that the collected responses were less concentrated around the mean [57]. The study was conducted in accordance with the Declaration of Helsinki and approved by the Institutional Review Board of King Faisal University (number: KFU254381, date of approval 5 January 2025). Ethical standards were strictly integrated into the data collection process. Additionally, all participants were provided with explicit explanations detailing the study’s objectives and written informed consent forms were obtained from all respondents prior to instrument administration. The collection and analysis of the data guaranteed full anonymity and confidential data aggregation.

### Data Analysis

Data analysis methods in structural research often combine multivariate techniques to validate measurement quality and test complex theoretical models. Specifically, data analysis is performed using Structural Equation Modelling (SEM) via AMOS 25 (IBM Corporation, Armonk, NY, USA) to evaluate hypothesized paths [58]. Furthermore, principal component analysis (PCA) with varimax rotation using SPSS version 25 (IBM Corporation, Armonk, NY, USA) is implemented to examine data representation quality and purify measurement scales. Normal distribution of the data was also checked and confirmed [58].

To ensure the psychometric rigor of the measurement model, internal consistency reliability, convergent validity, and discriminant validity were systematically evaluated. Internal consistency reliability was examined using Cronbach’s alpha coefficients [59]. Convergent validity was established by verifying that composite reliability values exceeded the recommended 0.70 threshold and average variance extracted (AVE) values surpassed the 0.50 threshold [60, 61, 62]. Furthermore, discriminant validity was assessed and confirmed by verifying that the square root of the AVE for each construct was greater than its bivariate correlations with all other constructs [62, 63].

To evaluate the indirect mediation effects within the structural model, a non-parametric bootstrapping procedure with bias-corrected confidence intervals was implemented using AMOS 25, rather than relying on standard normal-theory tests such as the Sobel test [64]. This bootstrapping approach was specifically selected to overcome the severe psychometric limitations and non-normality assumptions inherent in the traditional Sobel test, providing a more robust and precise assessment of indirect path significance [65].

### PCA and Purification of Measurement

PCA with varimax rotation was performed using SPSS version 25 to examine the quality of the representation. For the measured variables (work disengagement, burnout, and well-being at work), the Kaiser-Meyer-Olkin indices showed the following values (0.841, 0.879, and 0.775, respectively). Therefore, we conclude that our variables are suitable for factorial assessments. To verify reliability, the findings show that Alpha is excellent, displaying the following values (0.768–0.814 and 0.770). In addition, the specific p-value of all variables was zero; hence, the null hypothesis was rejected.

### Common Method Bias (CMB) Assessment

Since this study adopts a cross-sectional, single-source self-report questionnaire, it was important to ensure there is no potential threat of CMB. Therefore, Harman’s single-factor test was conducted prior to model estimation. The exploratory factor analysis with all substantive variables unrotated revealed that the first principal factor accounted for only 28.4% of the total variance, which falls well below the standard 50% threshold. Design and order of the items was counterbalanced. Furthermore, to rigorously test for CMB beyond Harman’s single-factor test—which is insensitive to moderate method variance—we applied the unmeasured latent method construct (ULMC) approach by adding a latent method factor to the measurement model. The model with the method factor did not fit significantly better than the baseline model without it, confirming that CMB does not invalidate our structural paths, though unmeasured method variance remains a minor limitation.

## Results

### Confirmatory Factor Analysis (CFA)

Prior to executing structural modelling, Table 2 summarizes the descriptive statistics for all retained items. Items, like D6 and D9, was removed during the initial psychometric purification phase due to an insufficient factor loading (<0.50) during exploratory factor analysis, which explains its omission from the final descriptive registry (see Fig. 2). Independent CFA fit indices for the reverse-adapted research disengagement scale confirmed strong psychometric properties (comparative fit index (CFI) = 0.997, Root Mean Square Error of Approximation (RMSEA) = 0.012). The CFA results showed that the factors were consistent with the data. The proportion of Chi-square to its degree of freedom (χ^2^/df) was 1.099, which is well below the conservative threshold of 3. The RMSEA index was 0.012, indicating an excellent fit. All baseline fit indices fell within highly acceptable ranges “normed fit index (NFI) = 0.964, Tucker-Lewis index (TLI) = 0.996, incremental fit index (IFI) = 0.997, and CFI = 0.997”. Skewness and Kurtosis indices indicated that all indicator distributions met the statistical assumptions for normal distribution.

**Table 2.** **Descriptive statistics**.

Variables		Minimum	Maximum	Mean	Standard deviation	Skewness	Kurtosis
Research–focused work disengagement							
	D1	1	5	4.08	0.817	–1.118	2.396
	D2	1	5	3.88	0.824	–0.403	0.089
	D3	1	5	4.07	0.818	–1.011	1.915
	D4	1	5	3.88	0.776	–0.514	0.644
	D5	1	5	4.01	0.797	–0.745	1.211
	D7	1	5	4.15	0.815	–0.934	1.162
	D8	1	5	4.17	0.805	–0.944	1.151
General burnout							
	B10	1	5	1.74	0.863	1.536	3.223
	B11	1	5	1.88	0.871	1.019	1.463
	B12	1	5	1.94	0.838	0.967	1.659
	B13	1	5	1.84	0.819	0.994	1.478
	B14	1	5	1.86	0.858	1.378	3.001
	B15	1	5	1.73	0.833	1.533	3.467
	B16	1	5	1.8	0.888	1.41	2.561
Well-being at work							
	W17	1	5	4.55	0.773	–2.326	6.569
	W18	1	5	4.53	0.782	–2.201	5.88
	W19	1	5	4.5	0.777	–1.994	4.883
	W20	1	5	4.18	0.926	–0.94	0.481

Model fit: (χ^2^ (122, N = 636) = 134,101, *p* < 0.001, normed χ^2^ = 1.099, GFI = 0.977, AGFI = 0.968, RMSEA = 0.012, Standardised RMR = 0.012, RFI = 0.954, IFI = 0.997, CFI = 0.997, TLI = 0.996, NFI = 0.964, PCFI = 0.795 and PNFI = 0.768). GFI, goodness-of-fit index; AGFI, adjusted goodness-of-fit index; RMSEA, root mean square error of approximation; RFI, relative fit index; IFI, incremental fit index; CFI, comparative fit index; TLI, Tucker-Lewis index; NFI, normed fit index; PCFI, parsimony comparative fit index; PNFI, parsimony normed fit index; RMR, root mean square residual.

**Fig. 2.**
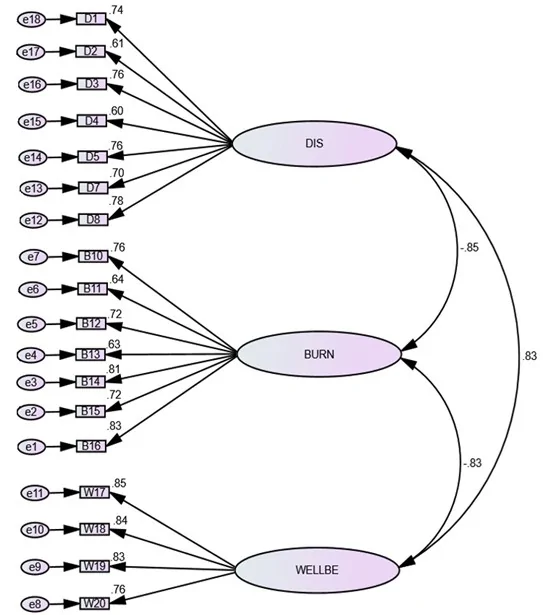
**The first order model**.

Convergent validity (CV) was checked by checking the composite reliability (i.e., above 0.7) and the AVE (above 0.5). As Table 3 shows, the CV was confirmed. Discriminant validity was also examined by verifying the square root of the AVE, which has to be higher than the relationship it shares with other constructs. As shown in Table 3, discriminant validity was confirmed though latent variable correlations exceeding 0.8 indicate substantial shared variance among constructs that must be interpreted with caution.

**Table 3.** **Convergent and discriminant validity**.

Factors/Items		Standard loading	Composite reliability	Average variance	Maximum shared	Average shared	1	2
				extracted	value	value		
Work disengagement (α = 0.768)			0.876	0.505	0.448	0.437	**0.711**	
	D1	0.74						
	D2	0.61						
	D3	0.76						
	D4	0.6						
	D5	0.76						
	D7	0.7						
	D8	0.78						
Burnout (α = 0.814)			0.89	0.538	0.448	0.446	–0.669*	**0.733**
	B10	0.76						
	B11	0.64						
	B12	0.72						
	B13	0.63						
	B14	0.81						
	B15	0.72						
	B16	0.83						
Well-being at work (α = 0.770)			0.892	0.674	0.444	0.435	0.652*	–0.652*
	W17	0.85						
	W18	0.84						
	W19	0.83						
	W20	0.76						

*p < 0.05. Bold diagonal numbers represent the average variance extracted (AVEs) for the related dimension.

### The SEM Results

The SEM was performed to assess the predictive associations of research-focused work disengagement on general burnout through well-being at work. The results showed that normed χ^2^ = 1.093, which was less than 3. Furthermore, all indices RMSEA = 0.012, NFI = 0.964, TLI = 0.996, IFI = 0.997, CFI = 0.997 and standardized RMR (SRMR) = 0.0236 were excellent (Fig. 3 and Table 4). The statistics revealed that work disengagement significantly predicated burnout (β = –0.509, *p* < 0.001). However, it significantly and positively predicated workplace self-actualization well-being (β = +0.830, *p* < 0.001). The latter significantly negatively affected burnout (β = –0.399, *p* < 0.001). Robustness of the structural model showed R^2^ coefficient of 0.539). This R^2^ value of 54% is remarkably high for behavioural and social science research [62], indicating that over half of the variance in lecturer burnout is accounted for by the interplay between research disengagement and teaching well-being.

**Fig. 3.**
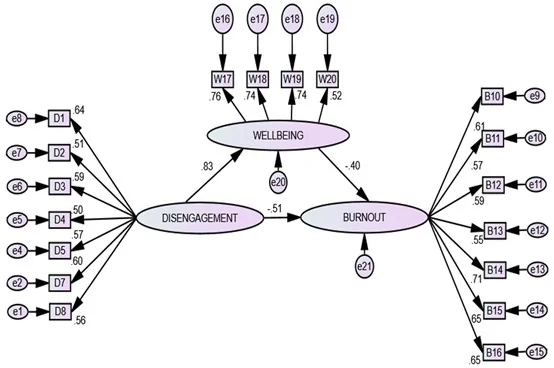
**The structural model**.

**Table 4.** **Result of the hypotheses**.

Hypotheses	β	CR value	Sig	Total effect	R^2^	Results
H1- D → B	–0.509	–5.274	***	–0.741		Supported
H2- D → W	0.83	12.921	***	0.83		Supported
H3- W → B	–0.399	–4.392	***	–0.399		Supported
H4- D → B Through W	–0.332		0.002			Supported
Burnout					0.539	

“Model fit: (χ^2^ (120, N = 636) = 131,193, *p* < 0.001, normed χ^2^ = 1.093, GFI = 0.978, AGF I= 0.968, RMSEA = 0.012, Standardised RMR = 0.0236, RFI= 0.954, IFI = 0.997, CFI = 0.997, TLI = 0.996, NFI = 0.964, PCFI = 0.782 and PNFI = 0.756)”. ***p < 0.001. CR, critical ratio; GFI, goodness-of-fit index; AGFI, adjusted goodness-of-fit index; RMSEA, root mean square error of approximation; RFI, relative fit index; IFI, incremental fit index; CFI, comparative fit index; TLI, Tucker-Lewis index; NFI, normed fit index; PCFI, parsimony comparative fit index; PNFI, parsimony normed fit index; RMR, root mean square residual.

While the structural pathways initially aligned with the sequential logic of traditional mediation models, the statistical significance of the indirect effect was assessed and confirmed exclusively using a non-parametric bootstrapping procedure (with 2000 resamples). This advanced approach was selected specifically to overcome the severe psychometric limitations of normal-theory tests, such as the traditional Sobel tests. By utilizing AMOS to generate bias-corrected asymmetric confidence intervals through bootstrapping, this study ensures rigorous validation.

**Table 5.** **Type of well-being at work mediation**.

The user-defined estimands					Mediation
Relationships	Est	Lower bounds (BC)	Upper bounds (BC)	Two tailed significance (BC)	
H4- D → W → B	–0.332	–0.503	–0.197	0.002	0.002 < 0.05

BC, bias-corrected.

Finally, maximum likelihood bootstrapping technique (utilizing 5000 resamples) within AMOS 25 confirmed a significant indirect effect of research disengagement on burnout through well-being (Indirect Effect (*ab*) = –0.332, *p* = 0.002). Because direct path remained statistically significant after controlling for mediator (β = –0.509, *p* < 0.001), results formally confirm that workplace self-actualization well-being exerts a significant partial mediating effect, supporting H4 (Table 5).

## Discussion

### Core Findings and Responses to the Research Questions

The main objective of current study is to examine the associations of disengagement from research on burnout directly and indirectly through the well-being of teacher-researchers, while answering the following question: To what extent do research-focused disengagement at work and the well-being of teacher-researchers associated with their professional exhaustion? Rather than a mere contradiction of established engagement frameworks [31], our findings reveal a contextual shifting of psychological resources. The results of this study confirmed that in environments where academic research lacks institutional reinforcement or evaluation, the investment of energy shifts entirely toward teaching, which explains why low research engagement paradoxically coexists with teaching-related well-being. Survey results supported our initial hypotheses by confirming that first hypotheses that research-focused disengagement at work significantly predicted general burnout (β = –0.509, *p* < 0.001), contradicting the conclusions of previous studies [19, 20, 21] and workplace self-actualization well-being (β = +0.830, *p* < 0.001), a result that challenges earlier research [30, 31, 32]. The latter significantly but negatively affects burnout (β = –0.399, *p* < 0.001), a result that is consistent with the research conducted by Rothmann [52]. Furthermore, the model reported that the relationship between work disengagement and burnout remained significant even after introducing well-being as a mediator (β = –0.332, *p* = 0.002 < 0.05). This allowed us to observe that well-being partially mediated the relationship between disengagement and burnout among teacher researchers.

### Explanations of Institutional Mechanisms

A plausible, contextually grounded explanation for these results lies in the institutional framework governing public universities in Tunisia. As outlined by Decree No. 93-1825, university lecturers face strict statutory mandates to actively engage in both teaching and scientific research. However, on an operational level, the institutional system lacks immediate, active disciplinary sanctions for research passivity, primarily enforcing career stagnation rather than termination or financial penalties. In this specific socio-institutional environment, we advance the interpretive hypothesis that selective disengagement from research acts as a constrained institutional adaptation. Rather than experiencing the chronic frustration of trying to secure scarce research resources or meeting publication demands under systemic constraints, faculty members deliberately withdraw their emotional and cognitive investment from the research domain. This defensive reallocation of energy protects them from the chronic stress that triggers burnout and preserves their psychological capital, explaining the significant negative path to exhaustion and the positive path to well-being. However, this contextual explanation must be treated with appropriate scientific caution because survey instrument did not directly operationalize or empirically measure “perceived administrative impunity” or statutory protection from sanctions rendering this an interpretive hypothesis requiring future mixed-methods validation.

### Evaluation of Alternative Explanations

To rigorously evaluate our findings, we must address several critical alternative hypotheses and potential methodological artifacts. First, we must emphasise that this study used a cross-sectional design and cannot support causal inferences; all reported paths should be interpreted as associations rather than causal effects, and alternative causal directions such as “burnout to disengagement” or “well-being to disengagement” (reverse causality) cannot be ruled out by cross-sectional data. Second, we must the possibility that the results reflect an artifact of our measurement strategy, specifically, the decision to adapt items from Schaufeli and Bakker’s [53, 54] UWES by explicitly reversing engagement items to focus on research detachment, scale may have captured a proactive, conscious state of psychological withdrawal rather than a passive, energy-depleted state of alienation. Third, an alternative explanation emerges from the clear overlap of referential domains across our measurement items. As illustrated in **Supplementary material**, our work disengagement items (D1–D8) were explicitly localized to capture withdrawal from the research domain, whereas our well-being items (W17–W20) explicitly captured emotional stimulation and professional accomplishment derived from the teaching domain. Consequently, the high positive path coefficient (β = +0.830) indicates a domain-shifting mechanism: as a lecturer psychologically detaches from the unrewarded and stressful demands of research, they actively redirect their remaining professional energy into classroom teaching, representing teaching-specific well-being rather than broad workplace satisfaction. Finally, high latent correlations (>0.8) present challenges to discriminant validity that must be acknowledged.

### Limitations and Future Research Opportunities

This study is no exception of research limitation. First, the specific nature of the cross-section design and sampling technique that the results obtained in the Tunisian university context may not be generalizable to other professions or other countries context without further validation. Therefore, to prove that disengagement at work leads to employee well-being, longitudinal studies need to be undertaken. Future studies should rectify this issue. Furthermore, in future research, it would be wise to test the possibility that social support may moderate the link between disengagement at work and burnout. For example, if a teacher-researcher experiences disengagement in their work but is supported by a permissive or even lenient laboratory director or by colleagues who share the teaching load, disengagement in this case would be a means of achieving well-being and avoiding the negative effects of burnout. Future research should integrate these findings into Social Exchange Theory (SET). Within the SET framework, the employment relationship is viewed as a series of interdependent, reciprocal interactions that generate obligations based on perceived organizational actions and rewards. Furthermore, content validity index expert ratings, reversed-item effect tests, potential content overlap inflation and self-selection bias from high non-publishing proportions (80%) and female representation (75%) represent notable methodological limitations that must constrain population generalizations.

In the Tunisian public university context, faculty members face legally mandated research requirements; yet they often experience a lack of institutional research infrastructure, funding, and immediate career incentives. According to SET, when employees perceive a fundamental imbalance in the socio-emotional or structural exchange—where the organization demands high-level scientific output but fails to provide the necessary supportive resources or reciprocal rewards—they may consciously choose to balance the exchange relationship by selectively withholding their cognitive and physical efforts from that specific domain.

### Implications of the Study

Regarding theoretical implications, the results of this research do not merely attempt to relativize existing theories on engagement, disengagement, and well-being; they contribute new knowledge by highlighting new counterintuitive relationships under COR theory that open new avenues of research. Theory must now incorporate the fact that, in harmful or even damaging work environments, or under high stress, disengagement is not necessarily a predictor of employee well-being but can act as a resource-preservation strategy. This finding suggests that disengagement can paradoxically maintain personal resources rather than weaken them. Furthermore, the results of this research encourage the scientific community not to limit itself to predefined assumptions (e.g., engagement is always beneficial) but to dig deeper to uncover the counterintuitive dynamics that may exist when the organizational system or governance is lacking.

This study cannot be meaningful or significant if it does not have managerial implications. Policymakers are responsible for establishing effective higher education governance and balancing reward/penalty systems. The results of this study highlight the problem of structural dysfunction. If teachers feel disengaged from research, this is because the pressure associated with it is poorly managed (unrealistic objectives, lack of time or resources, ineffective legislation, or simply a teacher-researcher who is unfit to conduct scientific research). In addition, decision makers are responsible for reviewing the evaluation and promotion criteria. Indeed, if scientific output is a source of burnout, the reward/penalty system is unbalanced. To this end, decision makers must re-evaluate teaching by motivating them to engage in scientific research and develop professional development programs for teacher researchers, focusing on scientific research methodology and academic publication strategies. Furthermore, it is up to public authorities to combat the inaction of teacher researchers by encouraging collaboration and collective responsibility. By breaking down their isolation, universities promote collective action and mutual support. Furthermore, the creation of working or research groups will have a positive impact on the sense of obligation and responsibility that teacher-researchers feel towards their peers and their specific organizations.

## Conclusions

This study provides a context-specific examination of the structural pathways linking domain-specific research disengagement, teaching well-being, and academic burnout among public university lecturers in Tunisia. The results of SEM showed a significant negative association exists between research-focused detachment and general professional exhaustion. The research showed that this relationship is partially mediated by the psychological fulfilment derived from daily teaching activities. While conventional organizational models generally dictate that role detachment erodes psychological capital, our path-analytic results suggest that under specific structural conditions, withdrawing from a highly stressful, unrewarded professional demand can be statistically associated with lower systemic burnout and higher localized well-being.

## Availability of Data and Materials

Data are available upon request from the corresponding author after the approval from the Ethical Committee.
