# Supplementary material for: Alleviating Exhaustion: The Mediating Role of Well-Being in the Relationship Between Research Disengagement and Burnout Among Tunisian University Lecturers
Source: Actas Esp Psiquiatr. 2026 Oct 15;54(5):1412–25. doi: 10.62641/aep.2419 (PMC13714457; doi:10.62641/aep.2419)
Supplement: Supplementary file 1 [file ActEsp-54-5-1412-1425-s1.zip › ActEsp-54-5-1412-1425.docx]

Supplementary Material: The research scale

| **Abbreviations** | **Item** |
| --- | --- |
| Work disengagement *(reversed to focus on research)* | |
| D1 | When it comes to research, I don't feel full of energy. |
| D2 | When it comes to research, I don't feel strong and vigorous. |
| D3 | I am not enthusiastic about scientific research work. |
| D4 | Research does not inspire me. |
| D5 | When I get up in the morning, I don't feel like doing scientific research. |
| D7 | I am not proud of what I do. |
| D8 | I am absorbed in my teaching work. |
| Burnout (*items cross-referencing research and teaching)* | |
| B10 | I feel exhausted at the thought of starting scientific research. |
| B11 | I feel exhausted in the morning at the thought of another day of scientific research. |
| B12 | When it comes to my scientific research, I find every hour of work tiring. |
| B13 | I don't have enough energy for my family and friends in my free time. |
| B14 | My teaching job is already emotionally exhausting. |
| B15 | My job frustrates me. |
| B16 | I feel exhausted by my teaching job. |
| Wellbeing at work (*items specific to the teaching domain)* | |
| W17 | I feel emotionally stimulated when it comes to teaching |
| W18 | I feel that I have a purpose in my work as a teacher |
| W19 | My work as a teacher is very important to me |
| W20 | I feel that I can continue to grow personally in my work as a teacher |
